# Revisiting Stereo Triangulation in UAV Distance Estimation

**DOI:** 10.3390/s26154798

**Published:** 2026-07-28

**Authors:** Jiafan Zhuang, Duan Yuan, Gaofei Han, Zhilang Weng, Rihong Yan, Weixin Huang, Wenji Li, Jie Xu, Zhun Fan

**Affiliations:** 1 Shenzhen Institute for Advanced Study, University of Electronic Science and Technology of China, Chengdu 610050, China; 2 Department of Electronic Engineering, College of Engineering, Shantou University, Shantou 515063, China; 3 Guangdong Laboratory of Artificial Intelligence and Digital Economy (SZ), Shenzhen 518000, China; 4 Center for Embodied Intelligence and Computer Vision, Shenzhen Loop Area Institute, Shenzhen 518000, China

**Keywords:** distance estimation, stereo triangulation, unmanned aerial vehicle

## Abstract

Distance estimation plays an important role for path planning and collision avoidance of swarm UAVs. However, the lack of annotated data seriously hinders related studies. In this work, we build and present a UAVDE dataset for UAV distance estimation, in which the distance between two UAVs is obtained by UWB sensors. During experiments, we observe that center-based stereo triangulation becomes unreliable in long-range UAV scenes. We show that this performance degradation is mainly caused by disparity errors introduced by practical UAV imaging conditions and amplified by long-range stereo geometry. To tackle this issue, we propose a novel position correction module, which predicts horizontal compensation offsets for the observed UAV centers under UWB distance supervision and applies them before stereo triangulation. Furthermore, to improve the robustness and generalization ability of position correction, we introduce a causal feature selection module into the position correction process. It adaptively selects correction-relevant causal feature channels and suppresses non-causal components caused by background interference, target appearance variation, and detection noise. We conduct extensive experiments on UAVDE. Our method achieves a significant performance improvement over a strong baseline (by reducing the relative difference from 49.4% to 8.6%), which demonstrates its effectiveness and superiority.

## 1. Introduction

Recently, the research of swarm unmanned aerial vehicles (UAV) has attracted increasing attention from researchers because of its wide applicability, such as environment exploration [1], autonomous search and rescue [2], target tracking and entrapping [3], flocking [4], task allocation [5], etc. To achieve an effective collaboration of swarm UAVs and avoid collisions, the reliable and accurate distance estimation of surrounding UAVs plays an important role.

Stereo-based distance estimation has been widely studied for many decades, especially in autonomous driving [6,7] and indoor robotic applications [8,9,10]. Existing methods [11,12,13,14] rely on matching each pixel densely between a pair of images captured by a stereo camera. Typically, a disparity map is predicted by using deep learning techniques and then is transformed into a distance map via stereo triangulation [15,16]. Benefitting from adequate and high-quality training data [17,18,19,20] annotated using LiDAR, these methods can achieve promising estimation performance.

Distance estimation has been widely used in other scenes, but it is rarely studied in UAV scenes because of two major challenges. (1) Data annotation in UAV scenes is difficult. As a common tool to obtain annotations for distance estimation, LiDAR can only produce sparse point clouds, which is adequate to capture various objects in urban [21] or indoor scenes [22]. However, it is unable to accurately scan a small UAV (e.g., 0.3 m) at typically long distance (e.g., 30 m) [23]. (2) Computation resource in UAV system is limited. Existing estimation methods rely on dense disparity prediction, which is computationally expensive. For example, a popular method AANet [24] can achieve nearly real-time performance (e.g., 16.13 FPS) on an NVIDIA V100 GPU (NVIDIA Corporation, Santa Clara, CA, USA). However, typical computing devices on UAVs, such as the NVIDIA Jetson TX2 and NVIDIA Jetson AGX Xavier (NVIDIA Corporation, Santa Clara, CA, USA), can only provide around 1/10 or even lower computation capability compared to an NVIDIA V100.

**Figure 1:**
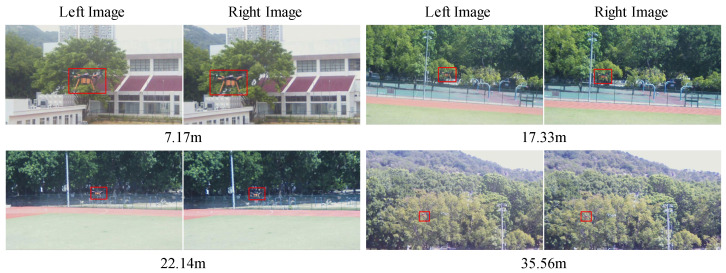
Samples from our presented UAVDE dataset. We collect thousands of UAV stereo images and annotate them with UAV bounding boxes and distances to the UAV center. Notably, only distance to the UAV center is collected via UWB sensors instead of collecting pixel-wise annotations on LiDAR, which is more economical and efficient for practical UAV applications. Best viewed in color.

Essentially, these two difficulties are both caused by the commonly adopted estimation paradigm, i.e., dense disparity prediction [25,26,27]. However, it is not necessary to predict the distance of each pixel in practical UAV scenes. As shown in Figure 1, the UAV of interest usually only occupies a small portion in the image. Moreover, considering the typically small size, it is adequate for path planning and collision avoidance only when provided with the distance to the UAV center, which is verified in previous successful applications [15,28,29].

Motivated by this observation, in this work, we build and present a dataset specifically for UAV Distance Estimation, named the UAVDE dataset. Different from existing datasets, we only annotate the distance between two UAV centers (i.e., the target UAV and the camera-shooting UAV) rather than densely annotating each pixel, as shown in Figure 1. Specifically, we equip ultra-wideband (UWB) sensors (Juzheng Technology, Shenzhen, China) near the center of each UAV, which can provide center-to-center distance annotations.

**Figure 2:**
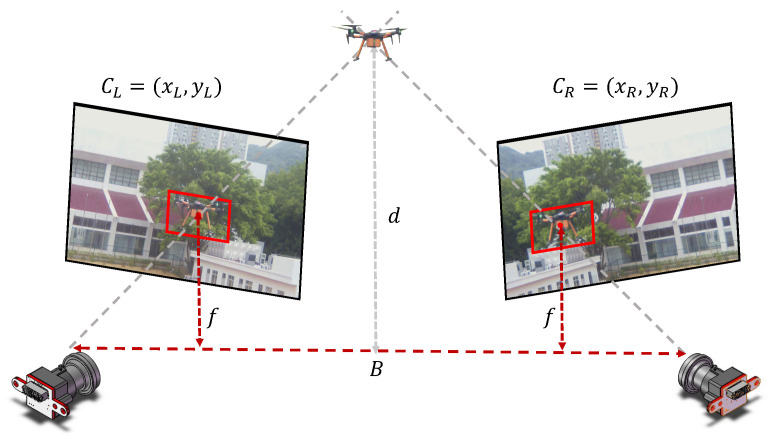
Stereo triangulation in UAV scenes. The centers of UAVs in stereo images can be obtained through UAV detection, which enables the computation of stereo triangulation to estimate the UAV distance. Here, *B* and *f* represent the baseline and focal length of the stereo camera, respectively.

**Figure 3:**
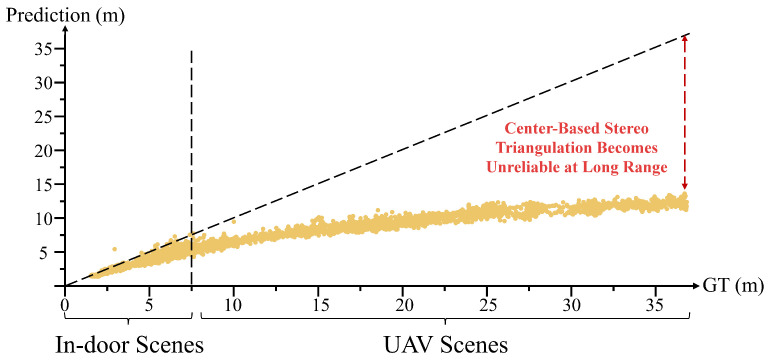
Upper-bound analysis of stereo triangulation in UAV scenes. The prediction is relatively accurate when the distance is small (e.g., less than 8 m), but as the distance increases, the prediction gradually deviates from the ground truth.

Based on our proposed dataset, existing distance estimation methods are not applicable due to the lack of dense distance annotations. Following a previous successful study [15], a practical solution is to conduct stereo triangulation based on UAV centers from stereo images, as shown in Figure 2. The UAV distance can be estimated as

(1) $$d=\frac{Bf}{x_{L}-x_{R}},$$

where *B* and *f* are the baseline and focal length of the stereo camera, respectively. The UAV centers can be simply obtained after UAV detection. To validate the effectiveness of this solution, we perform an upper-bound analysis by estimating distance with ground truth bounding boxes, which can remove the interference of the UAV detection performance. However, the experimental results reveal a clear performance degradation, as shown in Figure 3. The prediction is relatively accurate when the distance is small (e.g., less than 8 m), but gradually deviates from the ground truth as the target distance increases. These results indicate that standard center-based stereo triangulation becomes increasingly unreliable under practical long-range UAV conditions (e.g., greater than 20 m).

Why does stereo triangulation work well in indoor applications but become unreliable in UAV scenes? We argue that the main difficulty lies in the disparity error and its amplification under long-range UAV stereo geometry. In practical UAV scenes, the observed UAV centers in the left and right images may deviate from their ideal positions due to camera vibration, target motion, detector localization uncertainty, and small-target imaging. These factors introduce errors into the measured disparity.

Different from indoor scenarios, UAV distance estimation usually involves a limited stereo baseline and a much larger target distance. As a result, the true disparity becomes very small. In this case, even a minor pixel-level center deviation can significantly perturb the denominator of the triangulation formula and lead to a large distance error. This explains why stereo triangulation remains reasonable at short distances but gradually deviates from the ground truth in long-range UAV scenes, as shown in Figure 3.

This analysis suggests that the key to improving UAV distance estimation is to reduce the disparity error before triangulation. Therefore, we propose a Position Correction Module (PCM) to predict task-oriented horizontal compensation offsets for the observed UAV centers and compensate them before applying stereo triangulation.

Then, a natural question arises: Can such disparity errors be simply eliminated by careful camera calibration? Here, we provide estimation performance on different stereo images, which are calibrated via traditional methods [30,31] and recent learning-based methods [32], respectively. As shown in Table 1, the estimation performance is still unsatisfactory after calibration. This experiment demonstrates that the disparity error in UAV scenes is non-trivial and needs to be specifically addressed.

Based on the above analysis, we argue that reducing disparity errors before triangulation is critical for reliable UAV distance estimation, while the correction process should also avoid being affected by non-causal factors in practical UAV scenes. To tackle this issue, we propose a novel method named the Position Correction Module (PCM), which performs explicit compensation before stereo triangulation. The main idea is to predict horizontal compensation offsets from geometry-related position cues and apply them to the observed UAV centers before stereo triangulation. Although PCM can effectively alleviate the deviation issue, its offset prediction may still be affected by non-causal factors, such as background interference, target appearance variation, small-target imaging noise, and detector localization uncertainty. Therefore, to further improve the robustness and generalization ability of position correction, we introduce a Causal Feature Selection (CFS) module into the PCM. Specifically, CFS generates a differentiable feature-selection mask to identify correction-relevant causal feature channels and suppress non-causal components before offset regression. In this way, the PCM can focus more on stable geometric cues related to position deviation and reduce the influence of unstable scene-specific factors.

We experimentally evaluate the proposed method on the UAVDE dataset. The results validate the effectiveness of our correction method in UAV distance estimation, bringing a significant performance improvement. The contributions of this work are summarized as follows.

- We formulate the UAV distance estimation task and present a UAVDE dataset.
- We reveal that disparity errors amplified by long-range UAV stereo geometry are a major source of unreliability in center-based stereo triangulation under practical UAV conditions.
- We propose a novel position correction module (PCM) with a causal feature selection (CFS) mechanism to predict horizontal compensation offsets that reduce stereo-triangulation error. The CFS mechanism adaptively selects correction-relevant causal feature channels and suppresses non-causal components before offset regression, which improves the robustness of stereo triangulation compensation.
- We experimentally evaluate our proposed method on the UAVDE dataset, which demonstrates the effectiveness and superiority of our method.

The rest of this paper is organized as follows. We review the related works on UAV perception and stereo distance estimation in Section 2. Section 3 and Section 4 provide the details of our proposed dataset and approaches, respectively. Section 5 experimentally evaluates the proposed method. Finally, we conclude the work in Section 6.

## 2. Related Work

In this section, we review the literature relevant to our work concerned with visual perception in UAV scenes and common stereo distance estimation methods.

### 2.1. UAV Perception

Drones offer unique perspectives that traditional methods cannot achieve, thereby broadening the application of computer vision in aerial scenarios. This includes the enhancement of datasets [33,34] and tasks such as object detection [35], tracking [36], saliency prediction [37], and scene recognition [38] in UAV scenes. Specifically, Deng [35] introduced an end-to-end global–local self-adaptive network to tackle the issue of unevenly distributed and small-scale objects in drone-view detection. Li [36] achieved superior performance in robust object tracking by focusing on local parts of the object target. Fu [37] proposed a comprehensive video dataset for aerial saliency prediction. Bi [38] achieved impressive results by extracting key local regions based on a local semantic-enhanced ConvNet.

Although great progress has been made in some visual perception tasks, distance estimation in UAV scenes is still rarely studied, despite the fact that it is crucial for UAV applications. In this work, we formulate the UAV distance estimation task and present a well-established dataset to aid the corresponding research.

### 2.2. Classical Stereo Matching

For depth estimation from stereo images, many methods have been proposed in the literature which consist of matching cost computation and cost volume optimization. According to  [39], a classical stereo matching algorithm consists of four steps: matching cost computation, cost aggregation, optimization, and disparity refinement. As the pixel representation plays a critical role in the process, previous literature has exploited a variety of representations, from the simplest RGB colors to hand-crafted feature descriptors [40,41,42,43,44]. Together with post-processing techniques like Markov random fields [45], semi-global matching [11], and the Bayesian approach [46], these methods can work well on relatively simple scenarios, such as indoor scenes.

However, although most classical methods do not require a density map annotation, these methods often encounter challenges in real-world scenarios such as occlusions [47,48], diverse lighting conditions [49], and featureless regions [11], which are commonly observed in UAV scenes.

### 2.3. Learning-Based Stereo Matching

To deal with more complex real-world scenes, recent researchers leverage deep-learning techniques to extract pixel-wise features and match correspondences [25,26,27,50,51,52,53]. The learned representation shows more robustness to low-texture regions and various lightings [54,55,56,57,58]. Rather than directly estimating depth from image pairs, some approaches also sought to incorporate semantic cues and context information in the cost aggregation process [59,60,61,62] and achieved positive results.

Although learning-based methods can achieve significant improvement on estimation accuracy, they all rely on adequate and high-quality training data [6,62,63,64] densely annotated by LiDAR. However, LiDAR can not be applied in UAV scenes [23,65], and thus can not provide critical dense annotations for existing learning-based methods. Different from existing works, we build a new dataset for UAV distance estimation, which obtains the UAV distance based on UWB sensors instead of LiDAR. Based on this dataset, we revisit the stereo triangulation solution and discover the key issue of UAV distance estimation, i.e., position deviation, and propose a novel position correction method.

## 3. UAVDE Dataset

To aid the study of stereo distance estimation in UAV scenes, we present a novel UAV Distance Estimation (UAVDE) dataset. Here, we introduce the data collection process and the details of the annotation process.

### 3.1. Data Collection

**Figure 4:**
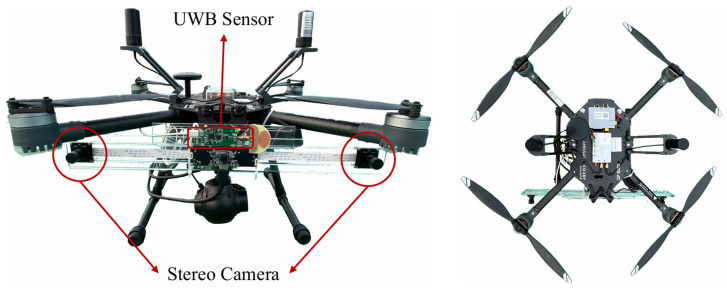
Illustration of the recording UAV. The UAV is equipped with a custom-assembled stereo camera system for image data collection and a UWB sensor for distance annotation collection.

To collect stereo images, we specifically use a recording UAV and a target UAV. The recording UAV is an AMOVLAB P600 (Chengdu Bobei Technology Co., Ltd., Chengdu, China) with a mounted custom-assembled stereo camera system and an ultra-wideband (UWB) sensor, as shown in Figure 4. Limited by the size of the UAV, the baseline of the stereo camera is set to 406 mm. The equipped lenses have a focal length of 12 mm, while the horizontal and vertical FOV are 22° and 18°, respectively. Furthermore, it is also equipped with an NVIDIA Jetson Xavier NX (NVIDIA Corporation, Santa Clara, CA, USA) as the computing platform, which is used for practical evaluation of distance estimation techniques. The target UAV is a DJI M200 (SZ DJI Technology Co., Ltd., Shenzhen, China) with a compact shape, which can achieve fast and steady flight performance and is suitable to serve as a detected UAV.

**Figure 5:**
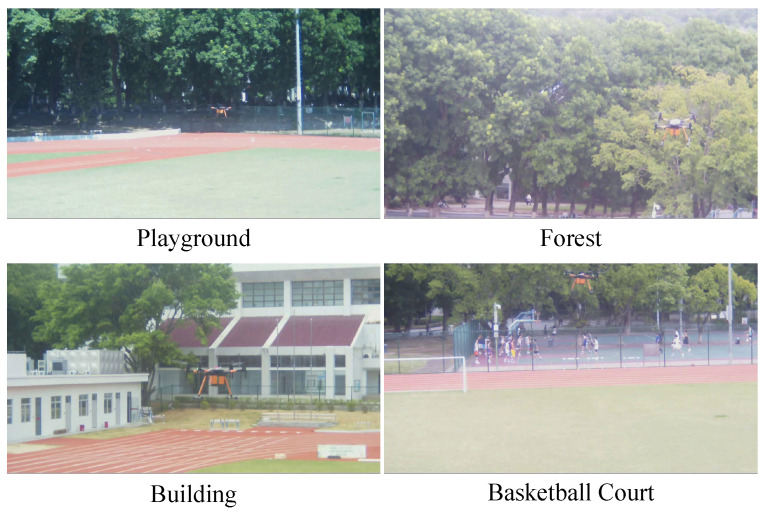
Illustration of typical UAV scenarios. We collect data in typical UAV scenarios, e.g., playground, forest, building and basketball court.

Motivated by practical applications, we select several typical scenarios for data collection, e.g., buildings, forests, playgrounds, and basketball courts, as shown in Figure 5. Based on these scenes, we collected 3895 stereo images in total, which are divided into training, validation, and evaluation subsets. To evaluate the adaptation ability to unseen scenes and prevent data leakage, the training, validation, and test subsets were divided according to different data-collection scenarios rather than by randomly splitting individual frames. Therefore, temporally adjacent frames collected in the same scenario were assigned to the same subset.

### 3.2. Data Annotation

In contrast to existing datasets with dense distance annotations collected by LiDAR, UAVDE focuses on the center-to-center distance between the recording UAV and the target UAV. This design is motivated by practical UAV applications, where the relative distance to the target UAV center is often sufficient for collision avoidance and formation control.

We use ultra-wideband (UWB) sensors to collect distance annotations. One UWB sensor is mounted near the center of the recording UAV, and another one is mounted near the center of the target UAV. The UWB system estimates their relative distance by measuring the signal travel time between the two sensors. During data collection, the stereo images and UWB ranging data were published as separate ROS topics and recorded concurrently in the same rosbag file. For each stereo frame, the UWB measurement with the nearest ROS timestamp was selected as its distance annotation. No temporal interpolation was performed during this timestamp-based association, and residual temporal misalignment was not further corrected. The UWB system operated at a sampling frequency of 40 Hz, with a ranging error of less than 10 cm. Data collection was started only after both UAVs had completed their flight maneuvers, reached a stable hovering state, and the image stream had become stable. Therefore, the relative motion between the two UAVs was limited during data acquisition. Combined with the 40 Hz sampling frequency of the UWB system, this helped reduce the temporal mismatch between the UWB measurements and the corresponding stereo frames. No additional synchronization mechanism, such as hardware triggering, was employed, and no Kalman filtering or other post-processing was applied to the raw UWB measurements. Compared with higher-precision measurement approaches, this UWB-based annotation process is lightweight, economical, and easier to extend to new UAV platforms and scenarios.

In addition to distance annotation, we manually annotate the bounding boxes of the target UAV in both left and right images. These bounding boxes are used for UAV detector training and for obtaining the target centers required by center-based stereo triangulation. It should be noted that UAVDE is designed for UAV center-distance estimation rather than dense depth prediction. Therefore, the UWB annotations directly match the target task of this work.

## 4. Method

### 4.1. Error Amplification in Stereo Triangulation

Before introducing the proposed position correction module, we first analyze why stereo triangulation is highly sensitive to center localization errors in long-range UAV scenes. Let $x_{L}$ and $x_{R}$ denote the ideal UAV center positions in the left and right images, respectively. The ideal horizontal disparity is

(2) $$s=x_{L}-x_{R}.$$

According to stereo triangulation, the target distance is given by

(3) $$d=\frac{Bf}{s},$$

where *B* and *f* denote the stereo baseline and focal length, respectively.

In practical UAV scenes, the observed UAV centers may deviate from their ideal positions due to vibration-induced dynamic extrinsic perturbations, target motion, detector localization uncertainty, and small-target imaging. In addition, the center of a detected bounding box may not coincide with the image projection of the physical UWB reference point, and this perspective-induced mismatch may vary with the target pose and viewing direction. Let $\delta_{L}$ and $\delta_{R}$ denote the center deviations in the left and right images. The observed centers can be written as

(4) $${\hat{x}}_{L}=x_{L}+\delta_{L},\;{\hat{x}}_{R}=x_{R}+\delta_{R}.$$

Thus, the observed disparity becomes

(5) $$\hat{s}={\hat{x}}_{L}-{\hat{x}}_{R}=\left(x_{L}+\delta_{L}\right)-\left(x_{R}+\delta_{R}\right)=s+\Delta ,$$

where

(6) $$\Delta =\delta_{L}-\delta_{R},$$

is the disparity error caused by center deviations in the two views. The distance estimated from the observed disparity is

(7) $$\hat{d}=\frac{Bf}{s+\Delta }.$$

The corresponding distance error can be derived as

(8) $$\hat{d}-d=\frac{Bf}{s+\Delta }-\frac{Bf}{s}=-\frac{Bf\Delta }{s(s+\Delta )}.$$

When |Δ|≪s, we have s+Δ≈s, and the absolute distance error can be approximated as

(9) $$|\hat{d}-d|\approx \frac{Bf}{s^{2}}\left|\Delta \right|.$$

Since s=Bf/d, this approximation can be rewritten as

(10) $$|\hat{d}-d|\approx \frac{d^{2}}{Bf}\left|\Delta \right|.$$

Similarly, the relative distance error is

(11) $$\left|\frac{\hat{d}-d}{d}\right|\approx \frac{d}{Bf}\left|\Delta \right|.$$

This analysis shows that the absolute error grows approximately with $d^{2}$, while the relative error grows approximately with *d*. Therefore, in long-range UAV scenes, even a small pixel-level center deviation can be amplified into a large distance estimation error. This explains why center-based stereo triangulation is much more unstable in UAV scenes than in short-range indoor scenarios. It also suggests that reducing the disparity error before triangulation is critical for reliable UAV distance estimation.

### 4.2. Position Correction Module

In this work, we mainly focus on alleviating disparity errors and propose a novel position correction module (PCM) to predict horizontal compensation offsets for the observed UAV centers before stereo triangulation. To this end, we first identify the factors that are closely related to the position deviation issue. Although standard camera calibration can largely compensate for systematic radial and tangential distortion, residual disparity errors may still arise from vibration-induced, frame-dependent perturbations of the stereo extrinsics. In addition, the center of a detected bounding box does not necessarily coincide with the image projection of the physical UWB reference point, and this perspective-induced mismatch may vary with the target pose, viewing direction, and image location. Therefore, the relative angle θ and radius *r* with respect to the image center are used as coarse image-location and viewpoint-related cues. As shown in Figure 3, the influence of disparity errors is also related to the distance of the target UAV. Since the distance is unavailable during inference, the width *w* and height *h* of the detected UAV bounding box are used as rough representations of the target scale and distance. Although PCM is applied to the left and right views separately, the predicted offsets $O_{L}$ and $O_{R}$ are jointly optimized through the stereo-triangulation equation under the UWB-supervised distance loss. Therefore, the final distance estimation does not rely solely on the monocular apparent-scale cues *w* and *h*.

**Figure 6:**
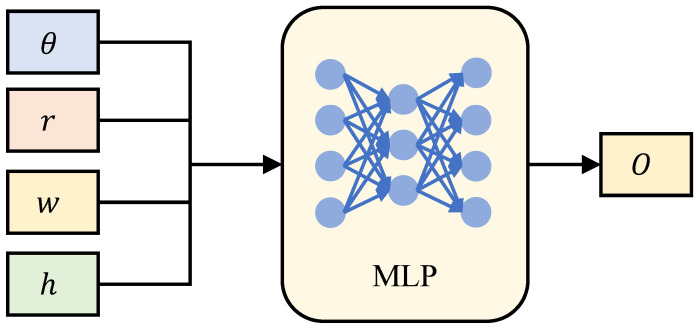
Illustration of Position Correction Module (PCM). Given several parameters describing the target UAV observation, we use a simple MLP to predict a horizontal compensation offset for stereo triangulation. Best viewed in color.

Based on the above analysis, we propose to use a 4-tuple {θ,r,w,h} to predict a horizontal compensation offset, as shown in Figure 6. In the position correction module, a simple multilayer perceptron (MLP) is adopted to perform prediction. Theoretically, any off-the-shelf MLP model can be adopted. Since the stereo triangulation is only related to the horizontal coordinate in Equation (1), the MLP is designed to regress a single offset variable *O* along the X-axis. For the rectified stereo image pairs considered in this work, the target distance is computed from the horizontal disparity between the left and right target centers. Therefore, only the X-axis offsets directly modify the disparity term in Equation (1), whereas a Y-axis offset does not directly affect the estimated distance. Nevertheless, the vertical target position is not discarded, because θ and *r* jointly encode the two-dimensional location of the target relative to the image center and are used to predict the horizontal compensation offset. This design assumes that major vertical misalignment between the two views has been removed through stereo calibration and rectification. Handling substantial residual vertical misalignment would require an additional rectification or correspondence mechanism and is outside the scope of the current method. Practically, PCM is conducted on the left and right images separately, resulting in $O_{L}$ and $O_{R}$. Based on the predicted offsets, we can compensate the computation of stereo triangulation as

(12) $$\tilde{d}=\frac{Bf}{\left(x_{L}+O_{L}\right)-\left(x_{R}+O_{R}\right)}.$$

During training, a simple $L_{2}$ loss is applied to the corrected distance $\tilde{d}$ and the ground-truth distance $d_{\mathrm{gt}}$.

(13) $$L_{\mathrm{pcm}}={\left(\tilde{d}-d_{\mathrm{gt}}\right)}^{2}.$$

Since ground-truth image-coordinate offsets are unavailable, PCM is not directly supervised to recover the true geometric displacement between the observed and ideal positions. Instead, the predicted variables $O_{L}$ and $O_{R}$ are optimized indirectly through the UWB-supervised distance loss in Equation (13). Therefore, they are interpreted as data-driven horizontal compensation offsets that reduce stereo-triangulation error, rather than estimates of the true image-coordinate offsets.

Notably, the training of the UAV detector and PCM is completely disentangled. Any off-the-shelf detector can be pre-trained on the UAVDE dataset and then used to produce 4-tuples {θ,r,w,h} for PCM training. During inference, PCM is simply attached to the detector to compensate the triangulation inputs before distance estimation.

However, directly using the full hidden representation of PCM may still introduce unstable and non-causal components, such as target appearance variation, background interference, and detector noise. Therefore, we further introduce a causal feature selection module to guide PCM to focus on correction-relevant causal feature channels.

### 4.3. Causal Feature Selection

**Figure 7:**
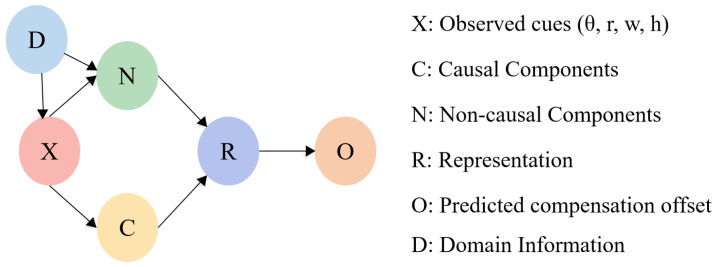
Structural causal model of the position-correction process. The observed cues X={θ,r,w,h} contain correction-relevant causal components *C* and domain-dependent non-causal components *N*, which form the representation *R* for predicting the compensation offset *O*.

**Figure 8:**
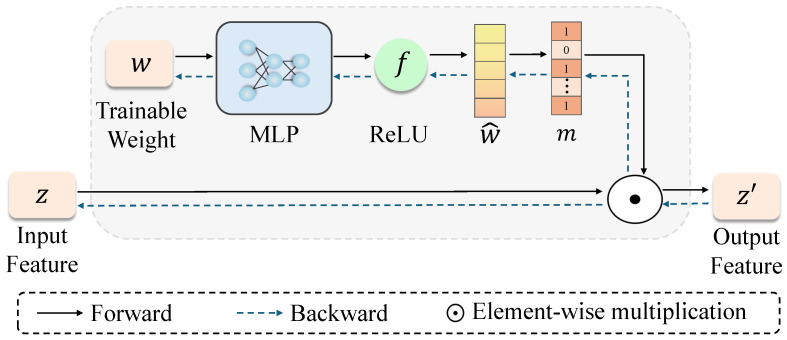
Illustration of the Causal Feature Selection (CFS) module. Given the hidden correction feature *z* from PCM, CFS transforms a trainable weight *w* through a lightweight MLP and a ReLU activation to obtain an intermediate variable $\hat{w}$. Then, $\hat{w}$ is converted into a differentiable binary-like mask *m*, which is applied to *z* through element-wise multiplication. The output feature $z^{\prime }$ preserves correction-relevant causal channels and suppresses non-causal components before offset regression.

Although the proposed PCM can effectively alleviate the position-deviation issue, its offset prediction may still be affected by unstable and non-causal factors in practical UAV scenes. To describe this issue, we introduce the structural causal model shown in Figure 7. The observed cues X={θ,r,w,h} contain correction-relevant causal features *C* and non-causal features *N*. The non-causal features are affected by domain information *D*, such as background texture, target appearance, small-target imaging noise, and detector localization uncertainty, which may vary across different scenarios. They are not stable causes of the true horizontal position offset. If these non-causal factors are encoded into the hidden representation *R* of PCM, they may introduce spurious correlations into offset regression and reduce the robustness of compensation offset *O*. Therefore, we further introduce a Causal Feature Selection (CFS) module to select correction-relevant feature channels and suppress non-causal components before offset prediction, as illustrated in Figure 8. It should be noted that CFS is a causally inspired feature-selection mechanism rather than a formal causal-inference method, and it does not perform causal identification or causal-effect estimation.

Given the 4-tuple X={θ,r,w,h}, PCM first maps it into a hidden correction feature:

(14) $$z=F_{\mathrm{pcm}}\left(X\right),$$

where $F_{\mathrm{pcm}}\left(\cdot \right)$ denotes the feature extraction layers of PCM and $z\in R^{C}$ denotes the hidden feature with *C* channels. Different channels in *z* may encode different correction factors. Some channels are related to stable geometric cues, such as image position and target scale, while other channels may be affected by non-causal factors. To perform causal feature selection, CFS generates a channel-wise mask $m\in R^{C}$ and applies it to the hidden feature:

(15) $$z^{\prime }=z⊙m,$$

where ⊙ denotes element-wise multiplication. The selected feature $z^{\prime }$ is then fed into the offset regression head:

(16) $$O=F_{\mathrm{reg}}\left(z^{\prime }\right),$$

where *O* denotes the predicted horizontal offset. In this way, the offset regression is encouraged to rely more on correction-relevant causal feature channels and less on unstable non-causal components.

The key step of CFS is to generate a differentiable binary-like mask. Specifically, we assign a trainable weight $w\in R^{C}$ to the CFS module and transform it into an intermediate variable:

(17) $$\hat{w}=\mathrm{ReLU}\left(F_{\mathrm{mask}}\left(w\right)\right),$$

where $F_{\mathrm{mask}}\left(\cdot \right)$ is a lightweight MLP. Then, the mask is generated as:

(18) $$m=\frac{{\hat{w}}^{2}}{{\hat{w}}^{2}+\epsilon },$$

where ϵ is a small positive constant. When $\hat{w}$ is close to zero, the corresponding mask value is close to zero and the feature channel is suppressed. Otherwise, the mask value is close to one and the corresponding feature channel is retained. This design enables end-to-end optimization without manually setting a hard threshold.

To guide CFS to identify stable correction-relevant feature channels, we introduce a feature consistency constraint based on Gaussian perturbation. For each input 4-tuple X={θ,r,w,h}, we construct an augmented input $X^{a}$ by adding Gaussian noise:

(19) $$X^{a}=X+\eta ,\;\eta ∼N\left(0,\sigma^{2}\right),$$

where η denotes the perturbation noise and σ controls the perturbation strength. The Gaussian perturbation is applied to the normalized 4-tuple, so that it only introduces mild variations to the observed position and scale cues without changing the underlying geometric relationship.

The original input *X* and the perturbed input $X^{a}$ are fed into the same CFS-enhanced PCM. After mask-based feature selection, we obtain the selected features $z^{o}$ and $z^{a}$ from the original and perturbed inputs, respectively. Since the correction-relevant geometric factors should remain stable under mild Gaussian perturbation, the selected features after CFS are expected to be consistent:

(20) $$L_{\mathrm{con}}={∥z^{o}-z^{a}∥}^{2}.$$

This constraint directly guides CFS to retain perturbation-invariant correction features and suppress perturbation-sensitive non-causal channels before offset regression.

The overall training objective is

(21) $$L=L_{\mathrm{pcm}}+\lambda_{\mathrm{con}}L_{\mathrm{con}},$$

where $\lambda_{\mathrm{con}}$ is the trade-off parameter.

### 4.4. Entire Pipeline

After introducing our proposed approaches, here, we provide an overview of the entire pipeline for distance estimation, which is shown in Algorithm 1. Specifically, RC represents the radian conversion, which can perform the conversion from the Cartesian coordinates into the polar coordinates.
**Algorithm 1:** UAV Distance Estimation with CFS-enhanced PCM**Input**: A stereo image pair $I_{L}$ and $I_{R}$**Output**: Estimated distance *d***UAV Detection****for** i∈{L,R} **do**$\;\;\lfloor \begin{array}{c} \;\{x_{i},y_{i},w_{i},h_{i}\}=\mathrm{Detector}(I_{i});\\ \;\{\theta_{i},r_{i}\}=\mathrm{RC}(x_{i},y_{i});\\ \;X_{i}=\{\theta_{i},r_{i},w_{i},h_{i}\}; \end{array}$**Position Correction with CFS****for** i∈{L,R} **do**$\;\;\lfloor \begin{array}{c} \;z_{i}=F_{\mathrm{pcm}}(X_{i});\\ \;{\hat{w}}_{i}=\mathrm{ReLU}\left(F_{\mathrm{mask}}\left(w_{i}^{\mathrm{cfs}}\right)\right);\\ \;m_{i}=\frac{{\hat{w}}_{i}^{2}}{{\hat{w}}_{i}^{2}+\epsilon ;}\\ \;z_{i}^{\prime }=z_{i}⊙m_{i};\\ \;O_{i}=F_{\mathrm{reg}}(z_{i}^{\prime });\\ \;{\tilde{x}}_{i}=x_{i}+O_{i}; \end{array}$**Stereo Triangulation**$d=\frac{Bf}{{\tilde{x}}_{L}-{\tilde{x}}_{R}}$;**return** *d*;

## 5. Experiments

### 5.1. Dataset

In this work, we build and present the UAVDE dataset to aid the study of distance estimation in UAV scenes. The training, validation, and evaluation subsets contain 2815, 541, and 539 stereo images at a resolution of 1280×720, respectively. Each sample contains a distance annotation and UAV bounding-box annotations on both left and right images. The validation subset is used for hyper-parameter and model selection. Following previous works [66,67,68], we adopt two commonly used evaluation metrics, i.e., Abs Rel and Sq Rel. All distances are expressed in meters. Accordingly, Abs Rel is dimensionless, whereas Sq Rel is measured in meters:

(22) $$\mathrm{Abs}\;\mathrm{Rel}=\frac{1}{N}\sum_{i=1}^{N}\frac{\left|d^{i}-d_{\mathrm{gt}}^{i}\right|}{d_{\mathrm{gt}}^{i}}.$$

(23) $$\mathrm{Sq}\;\mathrm{Rel}=\frac{1}{N}\sum_{i=1}^{N}\frac{{\left|d^{i}-d_{\mathrm{gt}}^{i}\right|}^{2}}{d_{\mathrm{gt}}^{i}}.$$

### 5.2. Implementation Details

In this work, we particularly adopt YOLOX-Nano [69] as the UAV detector due to its excellent trade-off between performance and computational efficiency, which is critical for computing device on UAVs. Specifically, we first pretrain the detector on the UAVDE dataset and follow the original training protocol, which achieves 55.3 mAP on the validation subset with a small inference resolution (352, 192) for efficiency. After pretraining, we fix the detector to generate 4-tuples {θ,r,w,h} for training our proposed modules.

For our PCM, we implement it with a lightweight MLP architecture, i.e., MLP-Mixer [70], which is a popular and effective MLP variant among various vision tasks. Since MLP-Mixer is designed for a sequence of embeddings, we can naturally feed our generated 4-tuple θ,r,w,h into MLP-Mixer sequentially. Benefitting from the mixing mechanism, MLP-Mixer can capture the internal relationship within the 4-tuple and extract hidden correction features. The proposed CFS module is inserted before the offset regression head to select correction-relevant causal feature channels and suppress non-causal components.

When training the PCM and CFS modules, we adopt an SGD optimizer with a learning rate of $10^{-3}$, a momentum of 0.9, and a weight decay of $10^{-3}$. Gradient clipping and a cosine learning-rate schedule with linear warmup are also adopted. For CFS training, Gaussian perturbation is applied to the normalized input tuple X=(θ,r,w,h) to construct augmented inputs for feature consistency learning. The consistency-loss weight, perturbation strength, and mask constant are set to $\lambda_{\mathrm{con}}=10$, σ=0.01, and $\epsilon =1\times 10^{-8}$, respectively.

Unless otherwise specified, all experiments involving PCM, PCM+CFS, and the other variants were conducted using the same fixed random seed and identical experimental settings to ensure fair and reproducible comparisons.

### 5.3. Performance Comparison

Since dense distance annotations from LiDAR are unavailable in UAV scenes, existing learning-based methods are not applicable. To demonstrate the superiority of our method, we make a comparison with two popular classical methods, i.e., ADCensus [71] and ELAS [46], which do not rely on dense annotations. We reproduce these methods according to their official codes on the proposed UAVDE dataset for fair comparison.

The performance comparison is shown in Table 2. Here, Baseline and Baseline* represent stereo triangulation on UAV detection results and annotated bounding boxes, respectively. From the results, we make the following observations. First, classical methods perform poorly on UAV scenes. They are affected by environmental interference, which results in error-prone disparity estimation. Second, baseline performs better than classical methods benefitting from the robustness of a deep learning model, i.e., the YOLOX. However, it still suffers from the position deviation issue. Annotated bounding boxes can only slightly improve the performance, which indicates that we can not tackle this issue by using a stronger detector. Third, our proposed method can significantly outperform its counterparts by over 40.8% improvement, because the CFS-enhanced PCM can jointly compensate position deviation and suppress non-causal correction features, which demonstrates its superiority and effectiveness.

### 5.4. Ablation Study

In this subsection, we conduct experiments to reveal the effectiveness of our proposed method.

#### 5.4.1. Effect of Components

Here, we conduct an ablation study to reveal the contribution of our proposed components, and the results are shown in Table 3. It can be seen that the baseline suffers from large estimation errors, with 0.494 Abs Rel and 6.818 Sq Rel on the test subset, which indicates that directly applying center-based stereo triangulation is unreliable in UAV scenes. After introducing PCM, the test Abs Rel is reduced from 0.494 to 0.121, and the test Sq Rel is reduced from 6.818 to 0.620. This result demonstrates that explicit position correction can effectively alleviate the position deviation issue before stereo triangulation.

Furthermore, by introducing CFS into PCM, the test Abs Rel is further reduced from 0.121 to 0.086, and the test Sq Rel is reduced from 0.620 to 0.262. This improvement shows that CFS can help PCM select correction-relevant causal feature channels and suppress non-causal components in the hidden correction features. Therefore, the CFS-enhanced PCM can provide more robust position correction and achieve better UAV distance estimation performance.

#### 5.4.2. Effect of PCM Input

To investigate the contributions of different input cues to PCM, we compare three input configurations: Full (θ,r,w,h), No-WH (θ,r), and WH-only (w,h). The bounding-box width and height are normalized by the image width and height, respectively. This input-ablation study uses a random seed different from that used in the main component-ablation experiments. Nevertheless, to ensure a fair comparison within this study, all three input configurations are trained and evaluated using the same fixed random seed and identical experimental settings.

As shown in Table 4, removing *w* and *h* increases the test Abs Rel by 111.64%, indicating that apparent-scale cues provide important complementary information for position correction. In contrast, using only *w* and *h* increases the test Abs Rel by 257.73% and produces a substantially larger Sq Rel, indicating more severe estimation errors. These results demonstrate that PCM benefits from jointly using image-position cues (θ,r) and apparent-scale cues (w,h). Because this input-ablation study and the main component-ablation experiments use different random seeds, the Full result reported in this table differs slightly from the PCM-only result reported in the component-ablation table.

#### 5.4.3. Analysis on Resource Consumption

Since the computing resource is highly limited on UAVs, the computation efficiency is critical for UAV distance estimation techniques. Here, we conduct an ablation study on the resource consumption of our proposed method, as shown in Table 5. Obviously, compared to the detector, the parameter and computation cost of our proposed PCM and CFS module is negligible due to the extremely light-weight design.

#### 5.4.4. Effect of CFS on Long-Range Samples

In this work, we introduce CFS to enhance the robustness of PCM on challenging samples, and its effectiveness is validated in Table 3. To provide a more intuitive analysis, we further evaluate the performance of PCM with and without CFS on samples of different difficulty levels, as shown in Table 6. The results lead to two observations. First, PCM consistently improves the distance estimation accuracy across all samples. Second, CFS provides additional improvements on challenging samples, particularly those with target distances greater than 20 m, where small localization deviations are more strongly amplified by stereo triangulation. These results demonstrate that CFS effectively improves the robustness of PCM under difficult long-range conditions.

#### 5.4.5. Generalization to Different Lenses

Since PCM and CFS are trained on data collected by predefined lenses, it is interesting to investigate the generalization ability of our method to testing data collected by different lenses. A promising generalization performance can make our method convenient to deploy on different UAVs efficiently after pretraining. Here, we collect a data subset via a 16 mm lens with a similar distance distribution to our test subset, which is different from the 12 mm lens used in building UAVDE. Different lenses change the imaging geometry, target scale, and the mapping between image position and center deviation, which may introduce lens-specific non-causal factors into offset prediction. As shown in Table 7, our method can still bring significant improvement compared to the baseline, demonstrating that CFS can help PCM focus on more stable correction-relevant feature channels. However, a performance degradation occurs compared to testing on in-distribution data (i.e., 12 mm), which remains as a direction for future research to further improve the generalization ability.

#### 5.4.6. Effect of Different UAV Detectors

To evaluate the adaptability of the proposed method to different UAV detectors, we conduct experiments with five YOLOX variants, including YOLOX-X, YOLOX-L, YOLOX-M, YOLOX-S, and YOLOX-Nano. These variants cover different model capacities and detection characteristics, allowing us to assess whether PCM+CFS can be combined with detectors of different scales. As shown in Table 8, PCM+CFS consistently reduces both Abs Rel and Sq Rel compared with the baseline across all YOLOX variants. This demonstrates that the proposed method is detector-agnostic and can effectively mitigate detection-induced localization errors, thereby enhancing the robustness of stereo triangulation for UAV distance estimation.

#### 5.4.7. Generalization to Different Target UAVs

**Figure 9:**
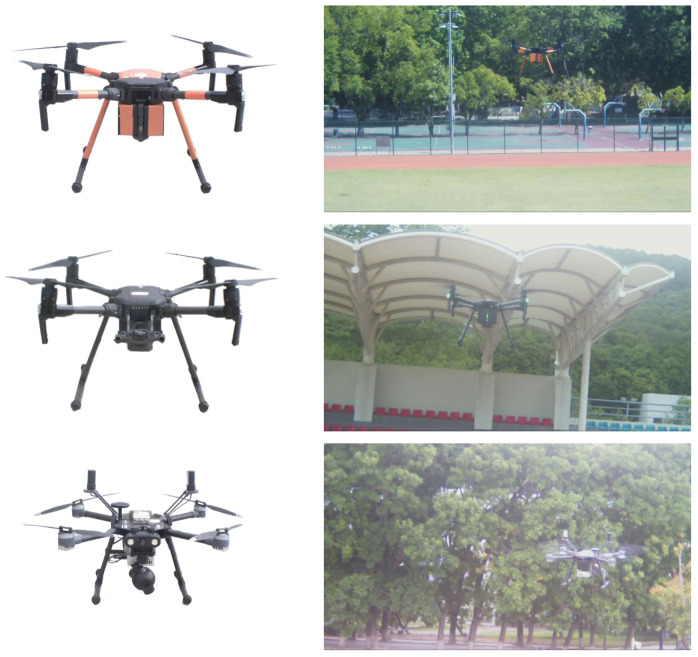
Illustration of different target UAVs. From top to bottom, the target UAVs are M200 Orange, M200 Black, and P600, respectively. Best viewed in color.

We further conduct a preliminary evaluation of the transferability of the proposed method across different target UAVs. Specifically, we collect two additional data subsets: one containing a UAV of the same type but a different color (M200 Black), and the other containing a UAV of a different type and physical size (P600), as shown in Figure 9. The UAV detector and the CFS-enhanced PCM are trained on the predefined target UAV (M200 Orange) in the UAVDE dataset and subsequently evaluated on these two out-of-distribution (OOD) subsets.

The results in Table 9 show that directly applying the original detector to unseen target UAVs results in relatively large distance estimation errors. When ground-truth bounding boxes are used, the CFS-enhanced PCM substantially reduces the errors for both unseen targets, indicating that detector quality is an important factor affecting cross-target transferability.

Under the ground-truth detection setting, PCM+CFS achieves lower Abs Rel on P600 than on M200 Black (0.108 vs. 0.220). A similar trend is observed with the finetuned detector (0.143 vs. 0.258). These results indicate that the proposed method does not necessarily perform worse on P600, despite its differences in target type, physical size, and structural characteristics. However, because the evaluation includes only two unseen target UAVs, the observed performance difference is insufficient to establish that the method is invariant to target size or other target-specific attributes.

To further examine the influence of detector performance, we use fewer than 50 samples from each unseen UAV type to finetune the detector. With the finetuned detections, the CFS-enhanced PCM achieves improved distance estimation performance on both targets. Overall, these results provide preliminary evidence of transferability to limited target variations. Nevertheless, evaluations involving more UAV types, physical sizes, appearances, and structural characteristics are required to comprehensively assess cross-target generalization.

#### 5.4.8. Visualization on Correction Effect

**Figure 10:**
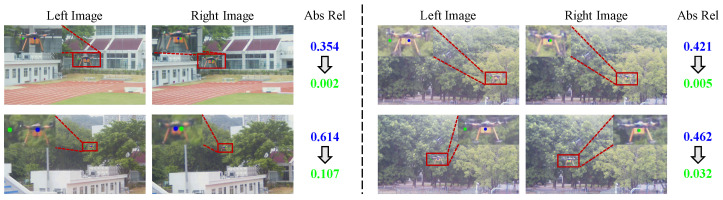
Visualization of correction effect. We visualize the observed UAV centers and the compensated centers produced by PCM+CFS on representative samples from the UAVDE test subset. Blue dots denote the observed centers, and green dots denote the compensated centers. The Abs Rel values show the distance estimation errors before and after compensation. The visualization demonstrates the improvement in distance estimation rather than the recovery of ground-truth image-coordinate positions. Best viewed in color and zoom in.

Here, we provide a visualization analysis on representative samples from the test subset of UAVDE, which provides an intuitive observation of the effect of position correction, as shown in Figure 10. Specifically, we visualize the observed UAV centers and the compensated centers produced by the proposed CFS-enhanced PCM, together with the corresponding improvement in Abs Rel. The results illustrate how the learned compensation changes the triangulation inputs and reduces the distance estimation error.

## 6. Conclusions

In this paper, we focus on the UAV distance estimation problem, which is practically important but rarely studied. To aid the study, we build a novel UAVDE dataset, and surprisingly find that standard center-based stereo triangulation becomes increasingly unreliable under practical long-range UAV conditions. We reveal that the main reason lies in disparity errors caused by practical UAV imaging conditions and amplified by long-range stereo geometry. To tackle this issue, we propose a novel position correction module (PCM) to predict horizontal compensation offsets for the observed UAV centers, which are applied before stereo triangulation. Furthermore, we introduce a causal feature selection (CFS) module into the position correction process to select correction-relevant causal feature channels and suppress non-causal components caused by background interference, target appearance variation, and detection noise. Extensive experiments validate the effectiveness and superiority of our method.

## Figures and Tables

**Table 1:** Performance comparison based on stereo images calibrated by different methods. The performance is still unsatisfactory since the position deviation issue cannot be simply tackled by careful camera calibration. Here, Abs Rel and Sq Rel are two common evaluation metrics for distance estimation.

Methods	Uncalibrated	Zhang [30]	Yan et al. [31]	Schöps et al. [32]
Abs Rel	0.478	0.468	0.469	0.471
Sq Rel	5.483	5.342	5.349	5.421

**Table 2:** Performance comparison on the UAVDE dataset. Baseline and Baseline* represent stereo triangulation on UAV detection results and annotated bounding boxes, respectively. Our method significantly outperforms other methods.

Method	Val		Test	
	Abs Rel	Sq Rel	Abs Rel	Sq Rel
ADCensus [71]	0.601	13.588	0.714	22.911
ELAS [46]	0.508	6.609	0.527	6.467
Baseline	0.490	6.716	0.494	6.818
Baseline*	0.483	6.550	0.488	6.712
Ours	**0.095 **	**0.581**	**0.086**	**0.262**

**Table 3:** Ablation study on our proposed components. Each component can bring performance improvement compared to the baseline.

Method	Val		Test	
	Abs Rel	Sq Rel	Abs Rel	Sq Rel
Baseline	0.490	6.716	0.494	6.818
+ PCM	0.148	1.014	0.121	0.620
+ PCM + CFS	**0.095 **	**0.581**	**0.086**	**0.262**

**Table 4:** Input ablation study of PCM. Different combinations of {θ,r,w,h} are evaluated to quantify the contributions of image-position and apparent-scale cues. The relative change is computed using Test Abs Rel with the Full configuration as the reference.

Group	PCM Input	Test		Abs Rel Change
		Abs Rel	Sq Rel	
Full	{θ,r,w,h}	**0.117**	**0.676**	+0.00%
No-WH	{θ,r}	0.247	1.478	+111.64%
WH-only	{w,h}	0.417	4.910	+257.73%

**Table 5:** Ablation study on resource consumption. We analyze the parameters and computation cost of our method. The actual speeds are evaluated on an NVIDIA Jetson Xavier NX device.

Module	Param (M)	FLOPs (G)	FPS
Detector	0.90	1.08	265
PCM	0.02	$3.144\times 10^{-6}$	1555
CFS	0.02	$4.800\times 10^{-8}$	7554
Detector + PCM	0.92	1.08	250
Detector + PCM + CFS	0.94	1.08	236

**Table 6:** Analysis of the effect on long-range samples. We analyze the effect of PCM and CFS on samples with different distance ranges on the validation and test subset of UAVDE. PCM can improve the performance consistently on different samples, while CFS further improves the robustness of position correction, especially for samples with distance larger than 20 m.

Method	Val		Test	
	<20 m	>20 m	<20 m	>20 m
Baseline	0.376	0.634	0.375	0.628
+ PCM	0.107	0.171	0.098	0.145
+ PCM + CFS	**0.088**	**0.120**	**0.084**	**0.088**

**Table 7:** Analysis on generalization to different lenses. We train the proposed modules using data collected with a 12 mm lens and evaluate them on data collected with 12 mm and 16 mm lenses. The 12 mm results are obtained on the complete UAVDE test set, whereas the 16 mm results are obtained on an independently collected generalization-test subset with a similar distance distribution.

Method	12 mm		16 mm	
	Abs Rel	Sq Rel	Abs Rel	Sq Rel
Baseline	0.494	6.818	0.430	5.090
+ PCM	0.121	0.620	0.199	**1.805**
+ PCM + CFS	**0.086**	**0.262**	**0.180**	3.186

**Table 8:** Robustness analysis with different YOLOX detectors on the UAVDE test set. We compare the baseline stereo triangulation and the proposed PCM+CFS method under different detector choices.

Detector	Baseline		PCM+CFS	
	Abs Rel	Sq Rel	Abs Rel	Sq Rel
YOLOX-X	0.490	6.768	0.073	0.207
YOLOX-L	0.490	6.774	0.071	0.217
YOLOX-M	0.492	6.841	0.073	0.247
YOLOX-S	0.492	6.837	0.078	0.272
YOLOX-Nano	0.494	6.818	0.086	0.262

**Table 9:** Analysis on generalization to different targets. We train PCM and CFS on the UAVDE dataset with a predefined target (M200 Orange) and evaluate them on different targets (M200 Orange, M200 Black, and P600) based on different UAV detections.

UAV Detection	Method	M200 Black	P600
Unfinetuned Detector	Baseline	2.551	2.399
	+ PCM	4.082	1.767
	+ PCM + CFS	**0.801**	**0.823**
GroundTruth Detection	Baseline	0.434	0.421
	+ PCM	0.355	0.389
	+ PCM + CFS	**0.220**	**0.108**
Finetuned Detector	Baseline	0.421	0.410
	+ PCM	0.343	0.376
	+ PCM + CFS	**0.258**	**0.143**

## Data Availability

The project repository is publicly available at https://github.com/Gaofei-Han/PCM (accessed on 22 July 2026). It currently contains the evaluation code, pretrained model weights, and selected test samples for reproducing the reported evaluation results. The complete training code and the UAVDE training data will be made publicly available in the same repository upon acceptance.
